# Vitamin D in the UK: an urgent call to redefine the threshold for deficiency

**DOI:** 10.3389/fendo.2026.1870408

**Published:** 2026-06-30

**Authors:** Samantha Christie, Suma Uday, Thomas R. Hill, Jonathan M. Rhodes, Christopher T. Sempos, Carrie H. S. Ruxton, Martin Hewison

**Affiliations:** 1 Greenwich Integrated Health, London, United Kingdom; 2 Birmingham Women’s and Children’s Hospital, Birmingham, United Kingdom; 3 Department of Metabolism and Systems Science, University of Birmingham, Birmingham, United Kingdom; 4 Human Nutrition and Exercise Research Centre, Population Health Sciences Institute, Faculty of Medical Sciences, Newcastle University, Newcastle upon Tyne, United Kingdom; 5 Department of Sport Science and Nutrition, Maynooth University, Maynooth, Ireland; 6 Molecular and Clinical Cancer Medicine, Institute of Systems, Molecular and Integrative Biology, University of Liverpool, Liverpool, United Kingdom; 7 Vitamin D Standardization Program, Havre de Grace, MD, United States; 8 Nutrition Communications, Cupar, United Kingdom

**Keywords:** deficiency, nutrition, osteomalacia, rickets, threshold, vitamin D, UK

## Abstract

ACT NOW Vitamin D is a multidisciplinary collective tackling persistent public health concerns in the UK, including widespread vitamin D deficiency, nutritional rickets in children, and osteomalacia across the lifespan. National Diet and Nutrition Survey data show significant declines in vitamin D intake - especially among lower intake percentiles - and rising rates of severe vitamin D deficiency (serum 25-hydroxyvitamin D <25 nmol/L) among people aged 4–64 years. Contributing factors include reduced meat, fish and seafood consumption, increasing obesity, and growing high-risk ethnic minority population groups. However, the current UK biochemical definition of vitamin D deficiency (<25 nmol/L), as defined by the Scientific Advisory Committee on Nutrition (SACN 2016), may underestimate the broader scale of population risk and obscure the clinical burden described above. This position statement therefore makes the case for UK policymakers to adopt a higher threshold for vitamin D deficiency than the current <25 nmol/L recommended by SACN 2016. Raising the threshold to <50 nmol/L would bring the UK into closer alignment with international and clinical practice frameworks and may enable a broader and preventive approach to surveillance of national vitamin D status. Such reclassification would also provide a stronger evidential basis for policy review aimed at improving population-level “Vitamin D Health”.

## 1 Background

There is increasing evidence of vitamin D deficiency and chronic nutrient insufficiency in the UK (1–5), particularly in high-risk groups: infants, children, pregnant women, ethnic minority populations (Black and South Asian populations), and individuals with health conditions, cultural practices, or lifestyle factors - such as obesity - that increase the risk of low vitamin D (25(OH)D) levels (6, 7). The most recognizable clinical manifestations of vitamin D deficiency - rickets and hypocalcaemic complications (8, 9) - remain stubborn UK health problems, with hypocalcaemia associated with risk of infant mortality (10, 11). While the persistence of vitamin deficiency in the UK has been recognised by UK health authorities including the Scientific Advisory Committee on Nutrition (SACN), Public Health England (PHE) and the National Institute for Health and Care Excellence (NICE), current guidelines have had only limited success in tackling this problem. Notably, the government has yet to publish a response to the 2022 Department of Health and Social Care’s (DHSC) call for evidence to improve UK Vitamin D Health, meaning that the next steps remain unclear.

In the UK, earlier vitamin D policy was shaped largely by the need to prevent rickets and osteomalacia and by the assumption that summer sunlight would provide sufficient vitamin D for much of the general population (12). As a consequence, in the 1990s, the Committee on Medical Aspects of Food and Nutrition Policy (COMA) did not recommend Reference Nutrient Intake (RNI) values for vitamin D, other than for those considered to be at high risk of vitamin D deficiency (12). However, in the early years of the 21^st^ Century, growing awareness of the potential dangers of sunlight exposure led to public health practices – sunlight avoidance and increased sunscreen use – that reduced the validity of the original COMA assumptions. In 2016 SACN published target Reference Nutrient Intake (RNI) recommendations aimed at improving vitamin D health in the UK. In their recommendations SACN concluded that 25 nmol/L 25-hydroxyvitamin D (25(OH)D) was the threshold of circulating vitamin D “below which risk of poor musculoskeletal health is increased and above which the risk is decreased at a population level” (13). This was then used to define the RNI for vitamin D that would ensure that the majority (97.5%) of the population remained above the threshold. The 25 nmol/L threshold continues to be the basis for UK public health recommendations for vitamin D intake, despite a lack of full alignment between this and the 25(OH)D target levels used in other clinical and international frameworks. For example, several authorities use 50 nmol/L (20 ng/mL) as a practical threshold for vitamin D sufficiency (14) or adequacy (see Section 4).

The 25 nmol/L threshold for 25(OH)D has also been incorporated into UK guidelines for the diagnosis and clinical management of vitamin D deficiency, despite SACN’s caution against this cut-off being used diagnostically (13). NICE Clinical Knowledge Summaries define vitamin D deficiency in adults as 25(OH)D <25 nmol/L, but with the caveat that levels >25 nmol/L but <50 nmol/L are still considered to be inadequate and are referred to as vitamin D insufficiency (15). This more complex three-tier approach to the diagnosis of vitamin D inadequacy is also reflected in UK strategies for clinical management of vitamin D deficiency that incorporate loading and/or maintenance supplementation regimens, depending on a diagnosis of vitamin D deficiency or insufficiency, and whether there are also specific clinical presentations (16).

The initial objective of ACT NOW Vitamin D is to make the case for consideration of a higher UK threshold for vitamin D deficiency (serum 25(OH)D <50 nmol/L), in light of international practice and the wider preventive health evidence summarised in this paper. We suggest that a 50 nmol/L threshold for vitamin D deficiency may better reflect clinical risk and support a more preventive approach to both the diagnosis and management of vitamin D deficiency. Such a threshold could also help to bring clinical and public health practice into closer alignment by providing a broader picture of the scale of vitamin D deficiency in the UK, thus prompting more effective strategies to address this persistent health problem.

## 2 Health implications of vitamin D deficiency in the UK

**Figure 1:**
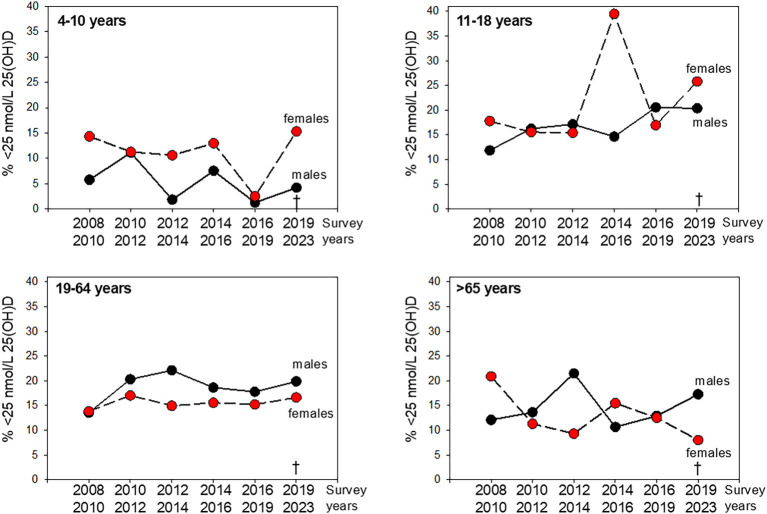
Percentage with serum vitamin D (25(OH)D) <25 nmol/L: NDNS years 1-15 (2008–2023). Data are shown for males (black circles) and females (red circles) in each age category (4-10, 11-18, 19–64 and > 65 years). Key: † Change in serum collection methodology (2019–2023).

**Figure 2:**
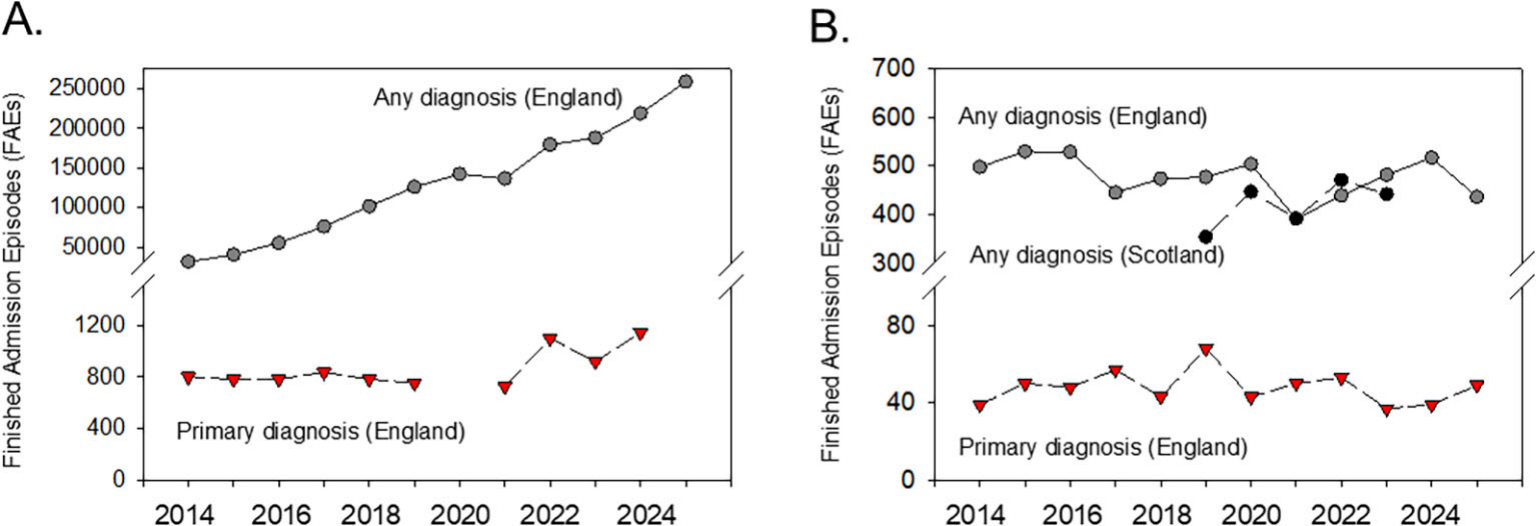
Reported cases of vitamin D deficiency **(A)** and rickets **(B)** in England based on primary and secondary diagnosis. Finished Admissions Episodes (FAE) coded for **(A)**: ‘vitamin D deficiency’ (E55) or **(B)**: ‘rickets’ (E550) with either primary or secondary diagnosis (any diagnosis, grey circles) or primary diagnosis alone (red triangles) in England for years ending 2014 to 2025. Black circles: Scottish rickets cases. Source: Hospital Episode Statistics (HES), NHS Digital, Freedom of Information request.

The 2025 National Diet and Nutrition Survey (NDNS), for 2019-2023, is the latest scientific assessment of micronutrients in the UK and provides a detailed picture of the changes in nutritional intake and serum 25(OH)D status across the life-course, relative to previous surveys going back to 2008 (4, 5). The most striking data from the 2025 report concern the percentage of individuals in each age group with serum 25(OH)D values <25 nmol/L; the deficiency threshold adopted by SACN in 2016 (**Figure 1**). The key observation from NDNS 2025 is that a significant percentage of the UK population continues to have serum 25(OH)D levels that are <25 nmol/L (10% of those aged 4–10 years, 23% aged 11–18 years, 18% aged 19–64 years and 12% >65 years) (5). Between the ages of 11 and 64 years the percentage <25 nmol/L 25(OH)D has increased steadily since 2008, although there was a 62% decrease in women over 65 with <25 nmol/L 25(OH)D, consistent with data from other countries showing higher use of vitamin D supplements in older subjects, notably females (17). The NDNS data are endorsed by recent NHS digital recorded instances of Finished Admissions Episodes (FAE) coded for ‘Vitamin D deficiency’ (E55) in England which showed that cases with <25 nmol/L 25(OH)D increased to 259,051 in 2025 (**Figure 2A**).

The clinical relevance of vitamin D deficiency in the UK continues to be debated, in part because a lack of definitive diagnostic criteria for osteomalacia means that vitamin D deficiency among adults is largely undiagnosed (18). Thus, the morbidity associated with vitamin D deficiency is likely to be underestimated. The following section will focus on the implications of vitamin D deficiency for health in infants and children.

### 2.1 Continued persistence of UK vitamin D deficiency rickets

NHS Digital data for ‘Vitamin D deficiency’ in England include many infants and young children (1,142 in 2024), suggesting continued risk of rickets, and FAEs coded for this disease (E550) persist at 400–500 cases annually (**Figure 2B**). These data refer to cases of rickets in England which represents 84% of the overall UK population. However, **Figure 2B** also shows comparative data from NHS Digital in Scotland (8.2% of the UK population) for 2018-2023. In the year 2022-2023, 442 cases of rickets (any diagnosis; primary or otherwise) were reported in Scotland - a relative rate of approximately 80 cases per million of the population compared to approximately 8.5 cases per million in England. Vitamin D deficiency is known to be more pronounced in Scotland due, in part, to its northerly geographical location.

The UK incidence of rickets has also been described with reference to the documentation of children within secondary healthcare with specific pre-defined clinical criteria. One study drew on 3500 UK paediatricians to report cases of either clinical rickets (leg deformity, swollen joints, abnormal calcium/parathyroid hormone (PTH)/phosphate biochemistry and serum 25(OH)D <25 nmol/L) or radiological rickets (abnormal metaphysis and serum 25(OH)D <25 nmol/L) (19). Over a 2-year period, 125 cases were identified with a further 36 cases recorded that did not meet stringent inclusion criteria (19). Using this strict clinical and biochemical definition, the overall incidence rate for rickets was reported as low (0.48 cases per 100,000 children annually under the age of 16 years). However, 105 of the cases were from Black or South Asian children, indicating a much higher incidence rate in these communities (5.0 and 2.75 cases per 100,000 children per year, respectively).

Given the rising proportion of dark-skinned immigrants in the UK, there is an urgent need to correct the disproportionality in rickets in Black and South Asian cohorts (6). Likewise, the incidence of rickets is much higher in infants and young children (<1 year or <2 years old): 1.90 and 3.49 cases per 100,000 children per year respectively compared to the older cohort aged >/=16 years (19). However, it is noteworthy that in this surveillance report, patients with active disease were excluded if their 25(OH)D levels were >25 nmol/l. Concerns have been raised about the large number of excluded cases in this report and the lack of data capture from GP records, leading to significant under-reporting of rickets in this survey (20). Earlier surveys of rickets in the UK between 1962–2011 showed a sharp increase in UK hospitalisation rates for rickets between 2000 and 2011 to levels that were the highest for five decades (21). Although these data have not been updated, it is reasonable to assume that rates of hospitalisation for rickets in the UK have not changed significantly.

While 25 nmol/L remains the SACN 2016 population risk threshold, the evidence for persistent rickets and its potential for co-morbidity, including fatalities (10), as well as cases of rickets with 25(OH)D >25 nmol/L, justify a 50 nmol/L population protective target to enable earlier preventive action. Critically, infants and young children with reported cases of clinically defined rickets represent only a small fraction of the vitamin D deficiency and musculoskeletal problems. Analysis of 29 diagnosed index cases of symptomatic vitamin D deficiency from tertiary referrals showed the wider familial impact of vitamin D deficiency and associated biochemical abnormalities (22). The median serum 25(OH)D level for the 29 index cases (12 nmol/L) was similar for the 29 mothers (13 nmol/L) and 68 siblings (19 nmol/L). These data highlight the broader health burden of vitamin D deficiency in families and communities, which is rarely diagnosed or recognised.

### 2.2 Hypocalcaemic cardiomyopathy and seizures

In a retrospective analysis of 160 young children who presented with ‘symptomatic vitamin D deficiency’ at the Royal Hospital for Sick Children Glasgow (2002–2008), the majority of cases presented with symptoms of rickets (bowed legs, impaired gait and swelling of wrists). Nevertheless, of clinical importance was the significant number of referrals due to low serum calcium-induced (hypocalcaemic) seizures (9), while the most serious and life-threatening complication of vitamin D deficiency, hypocalcaemic dilated cardiomyopathy, continues to be a clinical problem in UK infants (10, 11, 22, 23). Hypocalcaemia is a late clinical feature of vitamin D deficiency, due to compensatory mechanisms from secondary hyperparathyroidism that commence early in the disease process. Failure of these compensatory mechanisms leads to manifestations of hypocalcaemia which may include seizures in infancy and childhood and tetany in adolescence. Symptomatic hypocalcaemia due to vitamin D deficiency is more likely to manifest during rapid phases of growth such as infancy and adolescence (24). The British Paediatric Surveillance Unit survey of hypocalcaemic seizures due to vitamin D deficiency (September 2011 and 2013) reported a total of 91 cases of which 85% (n=77) were infants. This amounted to an annual incidence of 3.49 per million children (0–15 years), with the highest incidence in the South Asian population at 26.04 per million (8).

## 3 Factors affecting vitamin D status in the UK

### 3.1 Geographic and demographic effects on vitamin D status

Vitamin D can be obtained from dietary sources (see Section 3.2). However, the vast majority of circulating vitamin D (predominantly 25(OH)D) is derived from the epidermal action of ultraviolet (UV) light to generate vitamin D3, leading, in turn, to 25(OH)D3 after hydroxylation in the liver (25). Consequently, there are a wide range of factors that may influence UV light-generated 25(OH)D levels for any given individual. Geographical location is an important initial consideration. Epidermal photogeneration of vitamin D is less effective at northerly/southerly latitudes, and populations such as the UK experience pronounced seasonal peaks and troughs of vitamin D production (25).

Darker pigmented skin also requires more photons of UV light to generate vitamin D (26). This has less impact on vitamin D synthesis for individuals living in and around the equator, but in Northern European countries such as the UK, photogeneration of vitamin D is poor for the whole population for much of the year, and this will be exacerbated for individuals with darker skin pigmentation or among those not able to expose sufficient skin to UV light for medical or cultural reasons. Recent data from the UK Biobank assessing almost 450,000 adults aged 40–69 years, showed that 55% of men and women had 25(OH)D <50 nmol/L (2, 27). This decreased to 38-46% in Summer and Autumn but worsened to 68-71% during Winter and Spring. Prevalence of very low serum 25(OH)D (<25 nmol/L) was also clearly different between White (12%), Black (35%) and Asian (54%) participants (28). The over-arching message from these observations is that while vitamin D deficiency continues to be prevalent in all UK adults, it is a much more significant problem for Black and Asian communities.

The UK Biobank study also showed that serum 25(OH)D levels <25 nmol/L were observed in 20% of those with the highest index of multiple deprivation, compared with only 10% in those with the lowest index of deprivation (27). Similar differences were observed for participants defined as obese (19% <25 nmol/L) relative to those with a healthy weight (11% <25 nmol/L) (27), consistent with previous studies linking higher body mass index (BMI) with lower circulating levels of vitamin D (29, 30). The precise link between vitamin D and obesity continues to be debated, including possible mechanisms for adipose-driven suppression of 25(OH)D, and a causal role for low vitamin D in obesity i.e. a bidirectional effect (31). Nevertheless, it is now clear that the increasing prevalence of obesity in the UK is likely to be a critical factor in determining the ‘vitamin D health’ of the nation. BMI may be particularly important in both redefining targets for vitamin D sufficiency and the strategies required to meet these targets.

### 3.2 Declining dietary vitamin D intake in the UK

With the UK’s northerly latitude restricting dermal synthesis of vitamin D for much of the year, a sufficient dietary supply of the micronutrient is a primary public health concern. Consecutive European national food surveys report declining dietary vitamin D intakes over the last two decades (3–5, 32–38). Although dietary vitamin D inadequacy is not unique to the UK, sharp declines of up to 50% for vitamin D intake over the last two decades have been recorded in the UK at the lower intake percentile, and are a public health and nutritional safety concern when compared to other European countries including France, the Netherlands and Ireland (**Table 1**) (3–5, 32–38). Mean dietary intake of vitamin D as a percentage of the Reference Nutrient Intake (RNI) ranged between 25% and 34% RNI, indicating a significantly low dietary provision (RNI: 10 µg/day; latest surveys without methodological differences between assessments). The RNI is used here as COMA does not assign an Estimated Average Requirement (EAR) for vitamin D (12). Instead, UK individuals are recommended to achieve the RNI. The European Food Safety Authority (EFSA) does not provide an EAR but proposes an Adequate Intake threshold; 15 µg based on a 50 nmol/L target serum 25(OH)D (39). The finding of high risk of dietary deficiency in relation to the RNI extended across all intake percentiles among the selected European countries, aside from the highest intake percentile for UK men. Here, the RNI was just reached (p97.5; 102% RNI). The relationship between insufficient dietary intakes in relation to RNI has been repeatedly observed over the last fifteen years or so across Europe (40–47).

**Table 1:** Changes in dietary vitamin D intakes across selected European countries: time-trends across percentiles from national dietary surveys (2003–2019) †.

Country	Survey reference	Subjects	# years between surveys	Lowest mean intake vitamin D µg/day (p2.5/p5) (previous survey); latest	% change lowest mean intake vitamin D/day (p2.5/p5)	Latest intake as % of target RNI (lowest mean) RNI = 10µg/day	Mean daily vitamin D intake (µg/day) (previous survey); latest		% change mean daily vitamin D intake	Latest intake as % of target RNI (mean) RNI = 10µg/day	Highest mean vitamin D intake (µg/day) p97.5/p95 (previous survey); latest	% change mean highest vitamin D intake p97.5/p95	Latest intake as a % of RNI (highest mean) RNI = 10µg/day
UK	NDNS 2003; NDNS 2019	Males	16	(0.8); **0.4**	-50	4	(3.7); **3.2**	-13.5		32	(9.2); **10.1**	10.9	102
UK	NDNS 2003; NDNS 2019	Females	16	(0.5); **0.3**	-40	3	(2.8); **2.6**	-7.1		26	(8.4); **7.7**	-8.3	77
IR	NSIFSC 1997; NANS 2010	Males	13	(0.9); **0.7**	-22	7	(3.2); **3.4**	6.0		34	(8.4); **8.5**	1	85
IR	NSIFSC 1997; NANS 2010	Females	13	(0.6); **0.5**	-17	5	(2.6); **2.8**	8		28	(6.5); **7.1**	9	71
NL	DNFCS 2012; DNFCS 2021	Males	9	(1.4); **1.2**	-14	12	(3.6); **3.2**	-13		32	(6.7); **6.0**	-10	60
NL	DNFCS 2012; DNFCS 2021	Females	9	(1.1); **0.9**	-18	9	(2.7); **2.5**	-7		25	(5.1); **4.9**	-4	49
FR	INCA2 2006; INCA3 2014	Males	8	N/A	N/A	N/A	(2.7); **3.4**	25.9		34	N/A	N/A	N/A
FR	INCA2 2006; INCA3 2014	Females	8	N/A	N/A	N/A	(2.4); **3.9**	20.8		29	N/A	N/A	N/A

FR, France; IR, Ireland; N/A, not assessed; NL, the Netherlands; p2.5, 2.5th percentile; p5, 5th percentile; p97.5, 97.5^th^ percentile; p95, 95^th^ percentile; UK, United Kingdom; #, number; † Food only data sets; excluding food supplements (3–5, 32–38).

Values for latest survey shown in bold font.

These findings also have important implications for vitamin D deficiency prevention strategy. Given the limited natural food supply of vitamin D and the consistently low dietary intakes observed in the UK, recommendations for individual supplementation alone are unlikely to deliver equitable improvement in vitamin D status at population level. Food fortification therefore warrants explicit consideration alongside supplementation. Although mandatory fortification of UK margarine with vitamin D was in place in the UK for decades, this policy was introduced primarily because vegetable oil-based margarine was a common substitute for the analogue food, butter, and therefore required nutritional equivalence rather than as part of a broader strategy to address low vitamin D status (48).

Another important UK and Ireland perspective on food fortification comes from intake–status modelling in healthy adults, which showed that an intake of ~9 µg/day was sufficient to maintain serum 25(OH)D concentrations above 25 nmol/L in 97.5% of adults, broadly consistent with the current UK RNI of 10 µg/day, whereas substantially higher intakes were required to maintain concentrations at or above 50 nmol/L in almost all adults, at around 26.1 to 31.0 µg/day depending on sunshine exposure (49). This reinforces the need for coordinated fortification and supplementation strategies if a more preventive 50 nmol/L threshold is to be achieved in practice (48, 50). An important comparator is provided by Finland, where fortification of liquid dairy products and fat spreads was introduced in 2002 and increased further in 2010. Across FINDIET surveys, mean vitamin D intake increased from 5 to 17 µg/day in men and from 3 to 18 µg/day in women between 2002 and 2012, while mean serum 25(OH)D concentrations in adults reached 63 nmol/L in men and 67 nmol/L in women in 2012 (51). This illustrates that food fortification, alongside supplementation, can support meaningful improvement in vitamin D intake and status at population level.

#### 3.2.1 Changes in vitamin D-rich food consumption

The UK’s sizeable declines in consumption of both meat and meat products (-39%) as well as fish and seafood (-29%) in the last two decades, have contributed to the fall in vitamin D intakes, alongside other essential micronutrients, and specific fatty acids (**Tables 2**, **3**) (3, 4, 52, 53). Although meat does not provide the most concentrated supply of vitamin D across food sources, it is the primary source of the micronutrient for UK and Irish adults relative to other food supplies, and is also known to provide the more biologically-active form of vitamin D i.e., the downstream hydroxylated metabolites, 25(OH)D3 and 1,25-dihydroxyvitamin D3 (1,25(OH)_2_D3 (5, 37, 54). It should also be recognised that while UV-exposed mushrooms have been reported to contain vitamin D, this is provided in the form of vitamin D2 (55), and randomised trials of UV-irradiated mushroom supplementation have not shown a consistent increase in overall serum 25(OH)D levels (56).

**Table 2:** Vitamin D content of selected foods (means).

Food	Vitamin D (µg/100 g)
Pilchards, canned in tomato sauce	14.0
Trout, rainbow, grilled (including skin)	9.6
Tinned salmon, pink flesh only	9.2
Mackerel, chilled/frozen, raw flesh only	8.0
Grilled salmon	7.1
Tinned tuna (in oil, drained)	6.0
Sardines, canned (in oil, drained)	5.0
Egg, chicken, whole raw	3.2*
Butter	0.9
Liver, lamb, cooked	0.8
Lamb chop, raw	0.8
Chicken, grilled breast (without skin)	0.3†
Cow’s milk, full-fat (summer)	0.03
Meat substitutes e.g. mycoprotein, tofu, tempeh	0
Fortified	
Cereal, malted flakes	8.3
Margarine, soft polyunsaturated	7.9
Egg, free-range, enriched, whole raw	4.0^Ψ^
Milk substitutes (mixed: oat seed, soybean, almond; coconut flesh etc)	0.9
Mushrooms (UV exposed)	5^Ψ^
Bread, white flour (flour fortification)	0.6-2.1^Ψ^

Total vitamin D values are based on the UK Composition of Foods Integrated Dataset (CoFID) methodology. Use of a conversion factor to estimate total vitamin D activity by assigning a 25(OH)D3 bioactivity equivalent of 5x vitamin D3 for animal-derived foods, not including fish and seafood or eggs (83–85). * Does not delineate between barn, free-range or organic eggs – value is a composite of all systems. † Chicken skin contains 24 µg/100 g. Ψ Data for some fortified foods taken from websites (e.g. Happy egg co, Sainsburys vitamin D mushrooms and various bread producers including Hovis, Kingsmill, Warburtons and Marks & Spencer) (52–54).

**Table 3:** Relative vitamin D contribution of food sources: intake and changes across selected European countries.

Country (National food survey dates)	Food source with highest contribution to dietary vitamin D	% contribution of total dietary vitamin D	Change in mean intake of food source; all adults % [intake g/day; latest survey]
NL (2008/10-2012/16)	Fats and oils	36	-9% [24 g; **22** g]
IR (1997/9-2008/10)	Meat and meat products	30	2% [179 g; **183** g]
FR (2006/7-2014)	Fish and seafood	31	-13% [31 g; **27** g]
UK (2003–2019)	Meat and meat products	34	-39% [161 g; **99** g]

FR, France; IR, Ireland; NL, the Netherlands; UK, United Kingdom (3–5, 32–38).

Values for latest survey shown in bold font.

## 4 Justification for a <50 nmol/L vitamin D deficiency threshold

In Europe, EFSA (39), the European Society for Paediatric Gastroenterology Hepatology and Nutrition (ESPGHAN) (57), the European Society for Clinical and Economic Aspects of Osteoporosis and Osteoarthritis (ESCEO) (58), the German Nutrition Society (59), and the Polish Multidisciplinary Group (60) have all set vitamin D adequacy thresholds at greater than 50 nmol/L. Aligned with the 50 nmol/L value further afield, are the US National Academy of Medicine (NAM, previously the Institute of Medicine) (61), Health Canada (62), the Australian and New Zealand Eat for Health Society (63), the World Health Organisation/Food and Agriculture Organization of the United Nations (WHO/FAO) (64) and, more recently, the US Endocrine Society also endorsed 50 nmol/L (14). Therefore, most major authorities anchor vitamin D adequacy around >50 nmol/L for skeletal outcomes. The notable exceptions to this is SACN which adopted a <25 nmol/L threshold (13).

Several organisations have defined vitamin D thresholds in the context of human disease. For example, ESCEO proposed a 75 nmol/L 25(OH)D threshold based on adverse patient outcomes (58). However, the 50 nmol/L 25(OH)D threshold proposed by NAM in 2011 was based on optimisation of bone and skeletal health. This latter strategy was also central to the original SACN guidelines (13). SACN 2016, within its remit, did not revise the threshold for serum 25(OH)D set in earlier Department of Health (DoH) guidelines (65). Instead, it stated that the existing 25(OH)D threshold of 25 nmol/L was “retained,” reflecting its judgement that “evidence overall suggested that risk of poor musculoskeletal health was increased at serum 25(OH)D concentrations below about 20–30 nmol/L”. The 25 nmol/L level retained by SACN derives from the earlier DoH report, in which the threshold level of 25(OH)D was primarily based on prevention of bone disease. As DoH (65) stated: “Plasma levels of 25(OH) vitamin D found in clinical rickets or osteomalacia range from undetectable to around 20 nmol/L, and a level of plasma 25(OH)vitamin D of 25 nmol/L has conventionally been used as a cut off for defining the lower limit of adequacy of vitamin D status”. However, SACN emphasised that 25 nmol/L is not a clinical diagnostic threshold, but a population risk marker for musculoskeletal outcomes.

While no single vitamin D threshold uniformly predicts all skeletal outcomes, converging epidemiological, physiological, and interventional evidence supports a pragmatic public health threshold of 50 nmol/L. Since 2016, assay standardisation has improved, and new evidence has led to improved granularity. It is now reasonable to consider whether a higher population deficiency threshold (i.e. <50 nmol/L) is warranted, while maintaining population safety. With this in mind, the following sections examine the rationale for redefining the threshold adopted by SACN from earlier DoH guidelines.

### 4.1 Prevention of rickets in children

Vitamin D was initially discovered as a cure for rickets, and eradication of this bone disease continues to be a primary objective of vitamin D recommendations around the world. Consequently, a key catalyst for revising the serum threshold for vitamin D is the continued persistence of rickets in the UK, with 482 cases reported in England alone in 2023 (**Figure 2B**). The number of cases has largely remained unchanged since SACN 2016 reported, (529 cases in 2015-2016). Although trends reflect implementation and uptake challenges among services, rather than evidence appraisal *per se*, the inability to eradicate rickets in the UK 100 years after the discovery of the curative effects of vitamin D is a sobering call to action for public nutritional health and clinical medicine.

SACN’s threshold of 25 nmol/L for serum 25(OH)D is underpinned by reports from the DHSC (65, 66) which, in turn, cite the Johns Hopkins University School of Medicine’s laboratory reference range for healthy serum 25(OH)D: 25–137 nmol/L (67). However, as others have highlighted, the clinical-appropriateness of the 25 nmol/L limit has been called into question in the light of findings by Arnaud et al. who compared 9 children suffering from vitamin D deficiency-rickets to healthy age-matched controls (68). Of those with rickets, 7 had serum 25(OH)D levels >25 nmol/L and up to c.50 nmol/L - i.e. up to 100% higher than the threshold for increased risk of deficiency set by SACN which may be indicative of low calcium intake. However, the reference range used by the DHSC in 1991 appears narrower than the original data, which has been questioned elsewhere (69) and warrants clarification.

In a recent systematic review, Cashman and colleagues assessed 120 studies involving children up to 4 years of age with radiologically confirmed rickets to define a threshold serum level of 25(OH)D associated with the prevention of rickets (70). Based on individual participant data from 930 children in the study, the mean and median serum 25(OH)D values associated with rickets were 29 and 23 nmol/L respectively. The authors then used a Youden Index to determine the intersection of sensitivity and specificity of 25(OH)D as a biomarker and proposed a threshold of 28 nmol/L to prevent rickets in the large majority of children. Nevertheless, 38% of rickets cases in the study were associated with 25(OH)D levels >30 nmol/L. This is consistent with data from previous studies but also suggests that there are caveats to the proposed 28 nmol/L vitamin D threshold from this publication.

The first of these caveats is that the proposed threshold for 25(OH)D was based on a specific cohort – i.e. an analysis of calcium-replete children with adequate dietary calcium intake. The threshold rose to 40 nmol/L when the wider study population was included in the assessment, i.e., including children with inadequate calcium intakes (70). This difference, in part, explains the higher 25(OH)D thresholds proposed by other organizations as outlined above. For example, NAM indicated that in the face of inadequate calcium, a threshold of 75 nmol/L 25(OH)D may be required (61). Another earlier study described the risk of developing rickets with different serum levels of 25(OH)D and calcium (71). In this investigation, the odds of developing rickets in children with the lowest calcium intake accelerated dramatically when serum 25(OH)D values were <62.5 nmol/L, whereas the risk of developing rickets in those with the highest calcium intake only increased <35 nmol/L 25(OH)D.

A key limitation of existing threshold frameworks is their reliance on assumptions of calcium adequacy, which are not met in large segments of the UK population. UK data are strongly influenced by ethnic variations in calcium intake, i.e. 42% of women of African/Caribbean origin, and 36% of women of Asian origin have calcium intakes below the Lower RNI (LRNI), while only 8% of White women had calcium intakes below the LRNI (72). Collectively, these observations suggest that age, ethnicity, and sex variations in calcium intake are important confounders for vitamin D efficacy and challenge the merit of basing current vitamin D thresholds on adequate calcium intakes. Indeed, the second caveat from the systematic review on rickets (70) is that many of the children included in the study were reported as having ‘dark skin’; further emphasising that ethnic variations in vitamin D metabolism or function are likely to impact threshold recommendations for the wider UK population.

### 4.1 Infants and young children

Despite the most serious manifestations of vitamin D deficiency occurring in infancy and early childhood, there is a dearth of studies evaluating vitamin D status and requirements in this age group. A recent systematic review and individual participant data meta-analysis of children up to the age of 4 years, determined a minimum serum 25(OH)D threshold based on the risk of having rickets in young children (73). Analysis of odds, sensitivities, and specificities for nutritional rickets at different serum 25(OH)D thresholds suggested a minimal risk threshold of around 28 nmol/L for children with adequate calcium intakes and 40 nmol/L for children with low calcium intakes. However, there are methodological limitations in this and other similarly constructed reports. Notably, in a 2021 study to assess the serum 25(OH)D requirements need to prevent rickets in Nigerian children, Sempos et al. emphasised that “Determining the vitamin D requirements to prevent nutritional rickets has been thwarted by inconsistent case definition, inadequate adjustment for calcium intake and other confounders, and 25(OH)D assay variability”. Thus, while there is general agreement that serum levels of 25(OH)D <30 nmol/L create a risk of rickets, this threshold was not validated for accuracy using currently accepted quality assurance programmes such as the National Institute of Standards and Technology and DEQAS. There is also an inverse correlation between serum 25(OH)D and dietary calcium and, thus, estimation of the odds of having nutritional rickets should address this (71).

In SACN 2016, childhood rickets was one of several musculoskeletal outcomes considered when setting the RNI for vitamin D and retaining the <25 nmol/L population deficiency threshold. By contrast, ACT NOW Vitamin D believes that eradication of rickets in the UK should be a primary consideration when defining a revised population deficiency threshold for vitamin D.

### 4.2 Parathyroid responses

Beyond the prevention of rickets in children, the effects of vitamin D on other aspects of public health continue to be debated, with potential thresholds varying from 46–60 nmol/L for outcomes such as falls, fractures, cardiovascular function, physical performance and BMI (74). The most well recognised circulating marker of vitamin D is PTH, with serum PTH tending to be elevated under conditions of 25(OH)D deficiency, and feedback suppression of PTH following vitamin D supplementation and elevation of serum 25(OH)D. The non-linear nature of this association has been used to determine optimal serum levels of 25(OH)D, with maximal suppression of serum PTH proposed as corresponding to 25(OH)D sufficiency (74, 75). The relationship between serum 25(OH)D and serum PTH concentrations shows considerable variation and reported 25(OH)D thresholds vary between 30 and 100 nmol/L (76). Nevertheless, this suggests that there is significant vitamin D bioactivity beyond the 25 nmol/L level adopted by SACN. Although there have been reservations about the precise importance of PTH in defining thresholds for vitamin D (77), the impact of vitamin D on circulating PTH provides a strong molecular and physiological rationale for an upward revision of the population threshold for 25(OH)D deficiency.

### 4.3 Extra-skeletal effects of vitamin D

Over the last twenty-five years, it has become clear that vitamin D has the potential to influence facets of human health beyond the skeleton. The diverse array of non-classical actions demonstrated for vitamin D include antiproliferative, antimicrobial and anti-inflammatory effects that support a role for vitamin D deficiency in the pathophysiology of a wide range of extra-skeletal conditions (78). While there have been many reports linking low serum 25(OH)D with increased risk of common cancers, and infectious and autoimmune diseases, data from vitamin D supplementation trials are less clear. Nevertheless, the recent Clinical Practice Guidelines for vitamin D by the Endocrine Society endorsed the use of vitamin D supplements in children to not only prevent nutritional rickets but also lower the risk of respiratory tract infections (14). Additionally, the new guidelines support the use of vitamin D supplementation for reproductive health, prevention of transition from pre-diabetes to diabetes and mortality in the elderly (14), suggesting that extra-skeletal benefits of vitamin D are likely to be a more prominent feature of future vitamin D guidelines. There is still considerable debate concerning the serum 25(OH)D levels associated with extra-skeletal benefits of vitamin D, but the assumption is that these will be higher than the levels required to protect against rickets/osteomalacia. For example, in the VITAL randomised control trial, protection against autoimmune disease was associated with 25(OH)D increasing from 29.8 ng/ml (74.5 nmol/L) at baseline to 41.8 ng/ml (104.5 nmol/L) after one year (79). It is also interesting to note a recent study of all-cause mortality that emulated the VITAL and D-health vitamin D supplementation trials using UK Biobank data (80). Predicted data from this large cohort revealed that a significant improvement in all-cause mortality was expected with vitamin D supplementation, but only for subjects with baseline 25(OH)D <50 nmol/L (80). Thus, a target serum 25(OH)D level of 50 nmol/L may support broader benefits of vitamin D beyond bone health.

## 5 Conclusions and actions: the roadmap for change

The ACT NOW Vitamin D collective urges public health policymakers to consider whether the UK vitamin D deficiency threshold, currently set at <25 nmol/L as defined by SACN 2016, remains fit for purpose, and whether a higher threshold of <50 nmol/L should now be adopted. The current threshold may not fully capture the burden of rickets, osteomalacia, and persistent deficiency across different population groups, and remains insufficiently aligned with a number of international and clinical practice frameworks. The need for revision of the vitamin D threshold is further reinforced by sharp declines in vitamin D intake among those with the poorest dietary micronutrient provision, with pronounced disparities in vitamin D deficiency among Black, Asian, and Minority ethnic groups and those with obesity. A higher deficiency threshold would therefore help to bring public health considerations into alignment with clinical diagnostic and management practice, while also better reflecting preventive health evidence summarised in this paper.

At the same time, we recommend that clinical practice and management of vitamin D deficiency should be revisited with the aim of simplification. One option would be to move from the current three-tier system of diagnosis to two, i.e. individuals are either deficient or not (>/=50 nmol/L). The current framework (deficient, insufficient, sufficient) can introduce clinical ambiguity, particularly given the growing body of evidence that individuals within the 25–50 nmol/L range remain at risk of adverse skeletal outcomes. While differentiated treatment approaches may still be needed, the current classification system can increase practice burden, contributing to variation in interpretation and thresholds for action. Reclassifying this intermediate group as deficient would provide a clearer, prevention focussed, trigger for intervention, supporting more timely and consistent management, particularly in high-risk populations who are disproportionately represented within this range. The three-tier system was partly intended to reflect interactions with calcium intake; however, as calcium status is rarely assessed in routine practice, this distinction has limited clinical utility. A simplified two-tier approach, with targeted evaluation of calcium intake in high-risk groups, may be more practical and clinically meaningful. Adoption of the 50 nmol/L threshold for vitamin D deficiency may have the additional benefit of reducing demand for routine analysis of serum vitamin D levels at a time when the cost effectiveness of vitamin D assays in public health has come under increasing scrutiny (81). A threshold of 50 nmol/L would enable much broader assumptions of vitamin D deficiency for specific population groups, notably ethnic groups with darker skin pigmentation, pregnant women, teenage girls, people with obesity and any individual with limited capacity for outdoor activity, particularly in Winter months. This would not negate the continued need for routine vitamin D analysis in the setting of symptomatic vitamin D deficiency but would reduce the broader logistic and financial burden currently required for more generalised investigation of vitamin D status.

From a population implementation perspective, adopting a higher vitamin D deficiency threshold would support more consistent public health messaging, streamline clinical pathways, and reduce regional variation in practice. It would also improve alignment between laboratory reporting, prescribing guidance and preventative strategies. As such, policy makers should prioritise updating national guidance, harmonising clinical and public health frameworks and supporting education for healthcare professionals to facilitate a coordinated, fully integrated transition. Collectively, these actions would strengthen prevention efforts, reduce health inequalities, and improve population-level vitamin D status, while aligning with UK government priorities on disease risk reduction and evidence-based practice in order to mitigate vitamin D deficiency as a significant, yet preventable public health burden in the UK. Any revision of the UK threshold should, however, be accompanied by policy-led, practical population-level delivery measures, including consideration of food fortification alongside targeted supplementation, if meaningful and equitable improvements in vitamin D status are to be achieved.

In seeking an upward revision of the threshold for vitamin D deficiency in the UK we recognise that an immediate consequence of this would be to increase the numbers of people defined as vitamin D deficient, rather than insufficient. As outlined above, we hope that this will then catalyse new initiatives to improve the vitamin D health of the nation, including possible food fortification programmes, that are more likely to achieve serum 25(OH)D levels above the new threshold. If these objectives can be achieved, then it is reasonable to anticipate an improvement to current NDNS data, notably lowering the % of individuals with serum 25(OH)D < 25 nmol/L shown in **Figure 1**. It would be important to then assess whether this was associated with decreased clinical presentation of vitamin D deficiency – are cases of rickets still present if there are significantly fewer children < 25 nmol/L? The broader health benefits of raising the target for serum vitamin D are less clear. Vitamin D supplementation trials for extraskeletal health have often failed with respect to primary end points (14). However, in most cases these trials have been carried out against a background of baseline vitamin D sufficiency, where any improvement in vitamin D status is less likely to exert significant effects on disease outcomes. In view of the substantial percentage of UK subjects of all ages who continue to present with serum vitamin D levels < 50 mmol/L, there are potential opportunities to observe more meaningful effects of improved vitamin D status. It is exciting to consider that a revised threshold for vitamin D deficiency, coupled with new strategies for meeting this new target, may help to shed light on the wider health benefits of vitamin D.

The broader question of what threshold should be used for optimal vitamin D is much more difficult to define and outside the scope of this proposal. Recent studies by Pittas and colleagues showed that the efficacy of vitamin D supplementation in preventing progression to Type 2 diabetes was strongly influenced by vitamin D receptor genotype (82). It may be that we will need to move away from using serum 25(OH)D thresholds as the only target for vitamin D and human health, towards more personalised, genotype-defined thresholds. In the meantime, we believe that a meaningful step forward is required in the UK to reduce the risk for vitamin D-deficiency disorders. A simple move to <50 nmol/L as the national threshold for defining vitamin D deficiency would be a straightforward and effective way to achieve this.
